# Comparative Analysis of Chemical Composition and Bioactivities of Volatile Oils From *Wurfbainia villosa* Fruit and Stem

**DOI:** 10.1002/fsn3.72226

**Published:** 2026-08-08

**Authors:** Yuancong Gu, Bangyu Lv, Xingrui Nian, Xinrui Xie, Xinhe Yang

**Affiliations:** ^1^ College of Food Science and Engineering, Guangdong Ocean University Yangjiang China; ^2^ Yangjiang Research Institute of Guangdong Ocean University Yangjiang China

**Keywords:** antibacterial activity, antioxidant activity, GC–MS, hypoglycemic activity, volatile oil, *Wurfbainia villosa* Lour

## Abstract

This study extracted volatile oils from the fruit and stem of *Wurfbainia villosa* Lour. (*W. villosa*) using steam distillation. Their chemical compositions were comparatively analyzed using gas chromatography–mass spectrometry (GC–MS). Their biological activities were evaluated through antioxidant (DPPH, ABTS radical scavenging, and total antioxidant capacity), hypoglycemic (α‐glucosidase and α‐amylase inhibition), and antibacterial (agar diffusion) assays. The extraction yields were 1.87% for the fruit volatile oil and 0.13% for the stem volatile oil. GC–MS analysis identified 46 compounds in the fruit volatile oil and 59 compounds in the stem volatile oil. The fruit volatile oil was primarily composed of (+)‐α‐pinene (17.45%), camphene (14.53%), (−)‐β‐pinene (14.93%), D‐limonene (14.66%), borneol (16.18%), and bornyl acetate (3.57%). In contrast, the stem volatile oil was dominated by γ‐terpinene (17.78%), p‐cymene (17.29%), (+)‐4‐carene (13.46%), (−)‐4‐terpineol (9.53%), (−)‐β‐pinene (9.60%), β‐phellandrene (7.25%), and D‐limonene (5.31%). The two volatile oils shared 27 common components, of which 19 were unique to the fruit and 32 were unique to the stem. Orthogonal partial least squares‐discriminant analysis (OPLS‐DA) identified key differential markers, including γ‐terpinene, borneol, p‐cymene, (+)‐4‐carene, D‐limonene, (−)‐4‐terpineol, β‐phellandrene, and (−)‐β‐pinene. The fruit volatile oil demonstrated substantially stronger antioxidant activity (based on the DPPH and ABTS radical scavenging and total antioxidant capacity assays) and higher inhibitory activity against α‐glucosidase and α‐amylase than did the stem volatile oil. Conversely, the stem volatile oil showed substantially greater antibacterial efficacy against *Escherichia coli* and *Staphylococcus aureus* . Exploratory correlation analysis suggested that these bioactivity differences might be associated with the distribution profiles of characteristic chemical components. This study revealed substantial differences in the chemical composition and bioactivity of volatile oils from the fruit and stem of *W. villosa* . The findings provide a scientific basis for the targeted development of these volatile oils as natural antioxidants, hypoglycemics agents, or antibacterials agents, depending on the plant part.

## Graphical abstract

Volatile oils were extracted from the fruit and stem of *Wurfbainia villosa* by steam distillation, with yields of 1.87% for the fruit and 0.13% for the stem. GC–MS analysis identified 46 compounds in the fruit oil and 59 compounds in the stem oil, with 27 common components, 19 unique to the fruit, and 32 unique to the stem. Bioactivity evaluation revealed that the fruit volatile oil exhibited stronger antioxidant (DPPH, ABTS, FRAP) and hypoglycemic (α‐glucosidase, α‐amylase) activities than the stem oil. In contrast, the stem volatile oil demonstrated superior antibacterial activity against *E. coli* and *S. aureus* . These bioactivity differences are linked to the distinct chemical profiles of the two plant parts. The findings support the targeted development of fruit oil as a natural antioxidant/hypoglycemic agent and stem oil as a green antimicrobial.

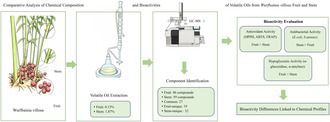

## 1 Introduction

Volatile oils (or essential oils) are complex mixtures of volatile, liposoluble secondary metabolites synthesized by aromatic plants. Their principal constituents, including terpenes, phenols, alcohols, and their derivatives, are typically stored in specialized structures like secretory epidermal or parenchyma cells (D'Addabbo and Avato 2021). Due to their diverse and complex chemical profiles, volatile oils exhibit a broad spectrum of biological activities such as antibacterial, antioxidant, anti‐inflammatory, and hypoglycemic properties, making them valuable natural resources (Fan et al. 2025; Zhang et al. 2025). Extracted primarily from various plant organs, these oils are characterized by high volatility and a rich aromatic profile. Recent research has extensively investigated the chemical composition and biological activities of plant volatile oils (Shang et al. 2026). Emerging studies continue to reveal diverse and potent functions, including antibacterial, antioxidant, anti‐inflammatory, hypoglycemic, neuroprotective, anxiolytic, and cognitive‐enhancing effects (Yohannis et al. 2026; Bungau et al. 2023).

*Wurfbainia villosa Lour*. ( *W. villosa* ) is listed among the “Four Major Southern Medicinal Materials” in the Pharmacopeia of the People's Republic of China (2020 edition). It is a perennial herb indigenous to Yangchun City, Guangdong Province, and holds National Geographical Indication status. With a cultivation history exceeding 1300 years, Yangchun's *W. villosa* is renowned for its superior quality domestically and internationally. Its core production area is the Jinhua Pit area of Panlong Village, Chuncheng Subdistrict, Yangchun City, Guangdong Province (Ma et al. 2024). *W. villosa* is rich in active compounds, including volatile oils, polysaccharides, polyphenols, and flavonoids. Modern pharmacological studies have demonstrated diverse biological activities, including antibacterial, antioxidant, antitumor, and gastroprotective effects (Peng et al. 2025). The primary edible and medicinal part is the fruit, in which volatile oils constitute its principal active fraction. Research indicates that its essential oil exhibits antioxidant and hypoglycemic properties (Wu et al. 2025), promotes intestinal health (Zeng et al. 2024), and inhibits neuroinflammation (Li 2023). Additionally, the fruit volatile oil has been reported to inhibit *Helicobacter pylori* , disrupt its biofilm formation, and delay its logarithmic growth phase (Huang et al. 2025). The leaves, an agricultural by‐product, contain volatile oils with antioxidant activity (Gu et al. 2025) and serve as a high‐quality green roughage resource (Yang et al. 2026). However, the stems remain underutilized, often being incinerated or discarded, leading to resource wastage and environmental pollution. Therefore, the high‐value utilization of *W. villosa* stem is of great scientific significance for reducing the potential ecological impact of agricultural waste and promoting the sustainable development of the *W. villosa* industry.

This study therefore focused on the fruit and stem of *W. villosa* . Volatile oils were extracted from both parts using steam distillation. Their chemical compositions were comparatively analyzed using gas chromatography–mass spectrometry (GC–MS). Their biological activities were further evaluated through assays for antioxidant capacity (including DPPH, ABTS radical scavenging, and total antioxidant capacity), hypoglycemic potential (including α‐glucosidase and α‐amylase inhibition), and antibacterial activity. The findings provide a scientific foundation for the high‐value development and comprehensive utilization of both plant parts.

## 2 Materials and Methods

### 2.1 Materials and Reagents

#### 2.1.1 Plant Materials

Mature fruit of *W. villosa* (hereafter “Fruit”) were sourced from the Jinhua Pit production area in Yangchun City, Guangdong Province, and procured from Yangchun Hengfeng Industrial Co. Ltd. Fresh stem (hereafter “Stem”) were harvested on March 31, 2025, from a cultivation base in Chuncheng Subdistrict, Yangchun City, Guangdong Province. Samples were immediately transported to the laboratory and stored at −20°C until use.

#### 2.1.2 Microbial Strains

Standard strains of *Escherichia coli* (CMCC 44103) and *Staphylococcus aureus* (CMCC 26003) were preserved in the Microbiology Laboratory of the College of Food Science and Engineering, Yangjiang Campus, Guangdong Ocean University.

### 2.2 Extraction of Volatile Oils

Following General Rule 2204 of the *Pharmacopeia of the People's Republic of China* (2020 Edition), volatile oils from the fruit and stem of *W. villosa* were extracted using steam distillation (Gu et al. 2025). Specifically, 50.0 g of fruit powder was placed in a round‐bottom flask with 500 mL of deionized water. Meanwhile, 50.0 g of fresh stem (moisture content 72%) was crushed and placed in a round‐bottom flask with 500 mL of deionized water. Each sample was subjected to distillation. The extraction yield (*Y*) was calculated according to Equation (1):

(1) $$Y\left(\%\right)=\left(m/M\right)\times 100\%$$

where *m* is the mass of the extracted volatile oil (g) and *M* is the dry weight of the plant material (g).

### 2.3 GC–MS Analysis of Chemical Composition

The chemical compositions of the volatile oils were analyzed by using gas chromatography–mass spectrometry (GC–MS).

#### 2.3.1 GC Conditions

An Agilent DB‐WAX capillary column was used. The inlet temperature was 250°C, with a constant flow rate of 1.2 mL/min in splitless mode. The oven temperature program was: hold at 40°C for 3 min; increase to 130°C at 3°C/min and hold for 3 min; raise to 190°C at 6°C/min and hold for 3 min; finally increase to 240°C at 10°C/min and hold for 3 min.

#### 2.3.2 MS Conditions

The mass spectrometer was operated with an electron impact (EI) ion source at 70 eV. The ion source temperature was 230°C. Data were acquired in full scan mode over a mass range of 50–500 m/z, with a 3 min solvent delay.

#### 2.3.3 KI Calculation

For retention index calculation, *n*‐alkane standards (C7–C40, ANPEL, 1000 mg/L in *n*‐hexane) were analyzed under identical GC–MS conditions.

### 2.4 Antioxidant Activity Assays

#### 2.4.1 DPPH Radical Scavenging Assay

The DPPH radical scavenging activity was assessed following a previously described method (Sun et al. 2022). Briefly, volatile oil sample solutions were prepared in anhydrous ethanol at concentrations of 1–5 mg/mL. For each test, 2 mL of sample solution was mixed with 2 mL of anhydrous ethanol and 2 mL of a 0.05 mmol/L DPPH ethanol solution. The mixture was vortexed, incubated in the dark at room temperature for 30 min, and the absorbance was measured at 517 nm as A_1_. A blank control was prepared by replacing the DPPH solution with 2 mL of anhydrous ethanol, and its absorbance was recorded as A_0_. Separately, 2 mL of the sample solution was mixed with 2 mL of anhydrous ethanol, incubated in the dark for 30 min, and the absorbance was measured and recorded as A_2_. Ascorbic acid served as the positive control. All assays were performed in triplicate. The radical scavenging rate (*S*, %) was calculated using Equation (2):

(2) $$S\;\left(\%\right)=\left[1-\left(A_{1}-A_{2}\right)/A_{0}\right]\times 100$$

#### 2.4.2 ABTS Radical Scavenging Assay

The ABTS radical cation scavenging activity was evaluated following a published method (Li et al. 2020). The ABTS^+^• working solution was prepared by mixing equal volumes of 1.48 mmol/L ABTS and 2.6 mmol/L potassium persulfate solutions, followed by incubation in the dark at room temperature for 12 h. The solution was then diluted with anhydrous ethanol to achieve an absorbance of 0.70 ± 0.05 at 734 nm (A_0_). For the assay, 2 mL of sample solution (1–5 mg/mL in ethanol) was combined with 2 mL of the ABTS^+^• working solution. After incubation in the dark for 15 min, the absorbance was measured at 734 nm and recorded as A_1_. Ascorbic acid served as the positive control. The scavenging rate was calculated in triplicate using Equation (3):

(3) $$\text{ABTS radical scavenging rate}\;\left(\%\right)=\left[\left(A_{0}-A_{1}\right)/A_{0}\right]\times 100$$

#### 2.4.3 Total Antioxidant Capacity (FRAP Assay)

The total antioxidant capacity was determined using the ferric reducing antioxidant power (FRAP) assay (Gao et al. 2025). The FRAP reagent was freshly prepared by mixing 10 mmol/L TPTZ (in 40 mmol/L HCl), 20 mmol/L FeCl_3_·6H_2_O, and 0.3 mol/L sodium acetate buffer (pH 3.5) at a ratio of 1:1:10 (*v*/*v*/*v*). FeSO_4_ standard solutions (0.2–1.4 mM, 1 mL each) were mixed with 4.5 mL FRAP reagent, incubated at 37°C for 30 min, and their absorbance was measured at 593 nm. A standard curve was plotted (*Y* = 0.7853*X* + 0.0169, *R* ^2^ = 0.999), where *Y* is absorbance and *X* is FeSO_4_ concentration (mM).

Volatile oils from *W. villosa* and its stem were dissolved in ethanol at concentrations of 1–5 mg/mL. Each solution (1 mL) was mixed with 4.5 mL FRAP reagent, incubated at 37°C for 30 min, and its absorbance was measured at 593 nm. FRAP values were calculated from the standard curve and expressed as mmol FeSO_4_ equivalent (1 FRAP value = 1 mM FeSO_4_). *V*_*c*_ served as a positive control. All assays were performed in triplicate.

### 2.5 Determination of Antibacterial Activity

The antibacterial activity of the fruit and stem volatile oils was evaluated against *E. coli* and *S. aureus* using the agar well diffusion method (Zhang et al. 2024). Briefly, 100 μL of a fresh bacterial suspension (adjusted to 10^6^ CFU/mL) was spread evenly onto sterile nutrient agar plates. After drying, 6‐mm‐diameter wells were aseptically punched into the agar. Subsequently, 10 μL of neat volatile oil was introduced into each well. Antibiotic discs containing ampicillin and penicillin served as positive controls. The plates were incubated at 37°C for 24 h. Antibacterial activity was quantified by measuring the diameter of the inhibition zones (including the well) using a digital Vernier caliper. All assays were performed in triplicate.

### 2.6 Hypoglycemic Activity Assays

The α‐glucosidase inhibitory activity was evaluated using a 96‐well microplate assay based on a published method with slight modifications (Song et al. 2024). Ethanol solutions of the fruit and stem volatile oils were prepared at concentrations of 1–5 mg/mL. The assay was performed as follows:

#### 2.6.1 Sample Well (A_1_)

A mixture of 40 μL of α‐glucosidase solution (0.5 U/mL in 0.1 M phosphate buffer, pH 6.8) and 40 μL of sample solution.

#### 2.6.2 Control Well (A_2_)

A mixture of 40 μL of phosphate buffer (0.1 M, pH 6.8) in place of enzyme and 40 μL of sample solution.

#### 2.6.3 Blank Well (A_3_)

A mixture of 40 μL of enzyme solution and 40 μL of phosphate buffer.

All wells were pre‐incubated at 37°C for 10 min. Subsequently, 20 μL of substrate solution (7.5 mmol/L p‐nitrophenyl α‐D‐glucopyranoside, PNPG) was added to each well, followed by incubation at 37°C for 30 min. The reaction was terminated by adding 100 μL of 0.1 M Na_2_CO_3_ solution. Absorbance was measured at 405 nm using a microplate reader. Acarbose served as the positive control. All experiments were performed in triplicate. The inhibition rate was calculated using Equation (4):

(4) $$\text{Inhibition}\;\left(\%\right)=\left[\left(A_{2}-A_{1}\right)/\left(A_{2}-A_{3}\right)\right]\times 100$$

#### 2.6.4 α‐Amylase Inhibition Assay

The α‐amylase inhibitory activity was determined using a 96‐well microplate assay based on the 3,5‐dinitrosalicylic acid (DNS) method (Elhafnaoui et al. 2026). Briefly, 100 μL of sample solution (1–5 mg/mL) was mixed with 100 μL of α‐amylase solution (9 U/mL in 0.02 M phosphate buffer, pH 6.9) in a microplate well. The mixture was incubated at 37°C for 10 min. Then, 100 μL of 1% (*w*/*v*) soluble starch solution (in the same buffer) was added as substrate, and the mixture was incubation continued at 37°C for 15 min. The reaction was terminated by adding 300 μL of DNS reagent, and the mixture was heated in a boiling water bath for 5 min. After cooling to room temperature, absorbance was measured at 540 nm.

Four reaction systems were set up for correction:

A_1_ (Sample): complete reaction system containing both sample and enzyme.

A_2_ (Control): system with sample replaced by buffer.

A_3_ (Background): system with enzyme replaced by buffer.

A_4_ (Blank): system with both sample and enzyme replaced by buffer.

Acarbose served as the positive control. All assays were performed in triplicate. The inhibition rate was calculated using Equation (5):

(5) $$\text{Inhibition}\;\left(\%\right)=\left[1-\left(A_{1}-A_{3}\right)/\left(A_{2}-A_{4}\right)\right]\times 100$$

### 2.7 Data Processing and Statistical Analysis

All experimental results are expressed as mean ± standard deviation (SD) of three independent replicates. For GC–MS data, chromatograms were processed, and components were tentatively identified by comparing their mass spectra with entries in the NIST 23 library. Compounds with a spectral similarity match factor ≥ 80 were retained for further analysis. The relative percentage content of each identified compound was calculated via peak area normalization. Data were analyzed using IBM SPSS 27.0. One‐way ANOVA (with *η* ^2^ for effect size) was used for multiple group comparisons, and the independent‐sample *t*‐test was used for two‐group comparisons. All data are shown as mean ± SD. Data visualization was performed using Origin software (version 2021). Multivariate statistical analysis, specifically orthogonal partial least squares‐discriminant analysis (OPLS‐DA), was conducted using SIMCA software (version 14.1) to identify key differential components.

## 3 Results

### 3.1 Comparison of Volatile Oil Yield From Fruit and Stem

**FIGURE 1:**
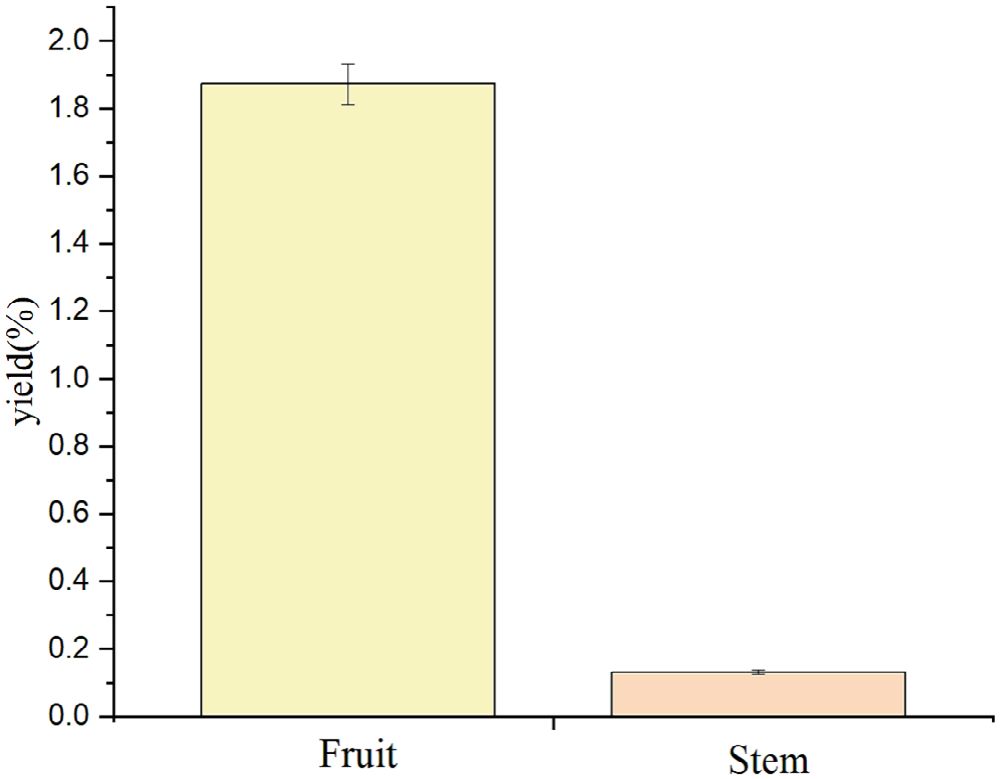
Comparison of the volatile oil yields between *W. villosa* fruit and stem.

The extraction yields of volatile oils from the fruit and stem of *W. villosa* obtained using steam distillation are shown in Figure 1. The fruit volatile oil yield was 1.87%, substantially higher than the stem yield of 0.13%. The fruit volatile oil yield was approximately 14.2‐fold higher than that from the stems. As the primary medicinal organ, the fruit likely accumulates higher levels of volatile oils for reproductive protection and bioactive metabolite storage. In contrast, the stems primarily serve as supportive and conductive tissues and typically synthesize and store lower levels of these secondary metabolites. This substantial difference in volatile oil content likely underpins the observed variations in their biological activities.

### 3.2 Analysis of Chemical Composition Types and Content in Volatile Oils of Fruit and Stem

**FIGURE 2:**
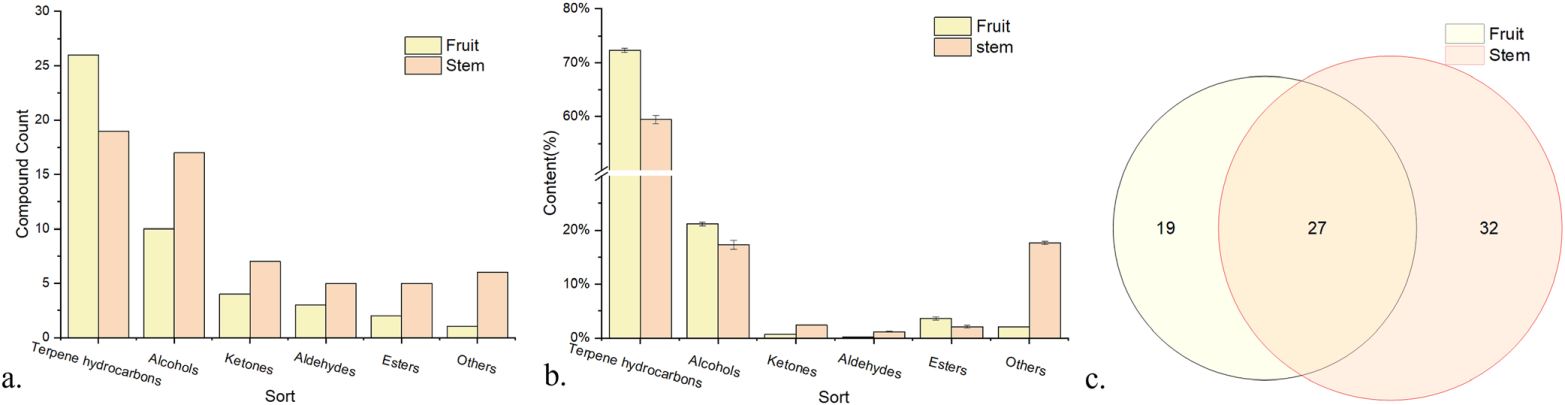
Differences in the chemical composition types and relative contents of volatile oils from *W. villosa* fruit and stem (a) composition quantity; (b) composition content; (c) Venn diagram.

To investigate differences in component types and relative content, Venn diagrams, component category analyses, and content difference plots were constructed for the volatile oils (Figure 2). A total of 46 and 59 components were identified in the fruit and stem volatile oils, respectively. In the fruit volatile oil, the main components were terpene hydrocarbons (26 compounds, 72.23%) and alcohols (10 compounds, 21.11%), followed by ketones (4 compounds, 0.71%), aldehydes (3 compounds, 0.22%), esters (2 compounds, 3.6%), and others (1 compound, 2.03%). In contrast, the stem volatile oil primarily consisted of terpene hydrocarbons (19 compounds, 59.42%) and alcohols (17 compounds, 17.30%), with ketones (7 compounds, 2.35%), aldehydes (5 compounds, 1.18%), esters (5 compounds, 2.06%), and others (6 compounds, 17.67%). Differences in the number and content of components, such as ketones, aldehydes, and esters, were also observed between the two oils (Figure 2a,b).

Based on Venn diagram analysis (Figure 2c) and Table 1, the stem volatile oil contained 32 unique compounds, including linalool (1.23%) and 3‐pinanone (1.40%) at relatively high levels. The fruit volatile oil contained 19 unique compounds, with relatively high levels of (+)‐α‐pinene (17.45%), bornyl acetate (3.57%), sabinene (2.35%), tricyclene (1.24%), and isoborneol (1.27%). Twenty‐six compounds were common to both volatile oils. These common compounds accounted for 71.61% of the fruit volatile oil and 89.91% of the stem volatile oil. Among terpene hydrocarbons, compounds exceeding 1% in the fruit volatile oil included camphene (14.53%), (−)‐β‐pinene (14.93%), α‐phellandrene (2.48%), γ‐terpinene (1.15%), and D‐limonene (14.66%). In the stem volatile oil, compounds exceeding 1% included (−)‐β‐pinene (9.60%), α‐phellandrene (1.11%), (+)‐4‐carene (13.46%), D‐limonene (5.31%), β‐phellandrene (7.25%), γ‐terpinene (17.78%), and allo‐ocimene (1.31%). Among alcohols, the major compounds in the fruit volatile oil were (−)‐4‐terpineol (1.71%) and borneol (16.18%), while in the stem volatile oil they were (−)‐4‐terpineol (9.53%), α‐terpineol (1.65%), and myrtenol (1.63%). Regarding other components, p‐cymene content reached 17.29% in the stem volatile oil, substantially higher than that in the fruit volatile oil (2.03%).

**TABLE 1:** Chemical composition of volatile oils from *W. villosa* fruit and stem.

No.	RT	Name	Molecular formula	CAS	KI	KI*	Fruit	Stem
Terpene hydrocarbons								
1	16.42	Tricyclene	C_10_H_16_	508‐32‐7	806	925	1.24% ± 0.03%	—
2	17.09	(+)‐α‐Pinene	C_10_H_16_	7785‐70‐8	821	932	17.45% ± 0.52%	—
3	18.78	α‐Fenchene	C_10_H_16_	471‐84‐1	858	950	0.13% ± 0.01%	0.36% ± 0.02%
4	19.19	Camphene	C_10_H_16_	79‐92‐5	867	943	14.53% ± 0.66%	0.78% ± 0.06%
5	21.70	Sabinene	C_10_H_16_	3387‐41‐5	923	974	2.35% ± 0.13%	—
6	22.00	Dehydrosabinene	C_10_H_14_	36262‐09‐6	929	956	0.13% ± 0.01%	0.46% ± 0.04%
7	23.14	3‐Carene	C_10_H_16_	13466‐78‐9	955	1011	0.01% ± 0.00%	—
8	23.67	(−)‐β‐Pinene	C_10_H_16_	18172‐67‐3	966	943	14.93% ± 0.28%	9.60% ± 0.30%
9	24.00	α‐Phellandrene	C_10_H_16_	99‐83‐2	974	1005	2.48% ± 0.06%	1.11% ± 0.08%
10	24.45	Pseudolimonene	C_10_H_16_	499‐97‐8	1139	1158	—	0.11% ± 0.02%
11	25.85	D‐Limonene	C_10_H_16_	5989‐27‐5	1010	1031	14.66% ± 0.63%	5.31% ± 0.08%
12	26.28	α‐Pyronene	C_10_H_16_	514‐94‐3	1187	1365	—	0.02% ± 0.00%
13	26.31	β‐Phellandrene	C_10_H_16_	555‐10‐2	1016	1031	0.82% ± 0.08%	7.25% ± 0.18%
14	26.61	1,3,8‐p‐Menthatriene	C_10_H_14_	18368‐95‐1	1021	1119	0.02% ± 0.01%	0.07% ± 0.00%
15	27.18	Bicyclo[3.1.1]hept‐2‐ene,3,6,6‐trimethyl—	C_10_H_16_	4889‐83‐2	1029	948	0.03% ± 0.01%	—
16	27.29	trans‐β‐Ocimene	C_10_H_16_	3779‐61‐1	1214	1229	—	1.51% ± 0.05%
17	28.06	γ‐Terpinene	C_10_H_16_	99‐85‐4	1042	1060	1.15% ± 0.09%	17.78% ± 0.15%
18	28.16	β‐Terpinene	C_10_H_16_	99‐84‐3	1043	1028	0.46% ± 0.05%	—
19	29.94	(+)‐4‐Carene	C_10_H_16_	29050‐33‐7	1069	1009	0.65% ± 0.14%	13.46% ± 0.16%
20	30.72	(E)‐4,8‐Dimethylnona‐1,3,7‐triene	C_11_H_18_	19945‐61‐0	1311	1312	—	0.02% ± 0.00%
21	30.81	γ‐Pyronene	C_10_H_16_	4724‐89‐4	1313	1406	—	0.02% ± 0.00%
22	33.96	Neo‐alloocimene	C_10_H_16_	7216‐56‐0	1128	1131	0.03% ± 0.00%	1.31% ± 0.04%
23	37.31	p‐Mentha‐1,5,8‐triene	C_10_H_14_	21195‐59‐5	1438	1453	—	0.11% ± 0.01%
24	39.55	γ‐Elemene	C_15_H_24_	29873‐99‐2	1468		—	0.11% ± 0.02%
25	40.37	Copaene	C_15_H_24_	3856‐25‐5	1221	1376	0.77% ± 0.27%	—
26	41.92	(+)‐epi‐Bicyclosesquiphellandrene	C_15_H_24_	54274‐73‐6	1243	1482	0.04% ± 0.02%	—
27	43.21	α‐Santalene	C_15_H_24_	512‐61‐8	1262	1420	0.10% ± 0.04%	—
28	44.20	Bicyclo[5.2.0]nonane,2‐methylene‐4,8,8‐trimethyl‐4‐vinyl—	C_15_H_24_	242794‐76‐9	1276	1407	0.05% ± 0.02%	0.11% ± 0.03%
29	46.69	δ‐EIemene	C_15_H_24_	20307‐84‐0	1355	1338	0.05% ± 0.01%	—
30	47.20	Aristolochene	C_12_H_24_	26620‐71‐3	1575		—	0.03% ± 0.01%
31	47.60	α‐Patchoulene	C_15_H_24_	560‐32‐7	1412	1457	0.06% ± 0.01%	—
32	48.12	Bicyclogermacrene	C_15_H_24_	24703‐35‐3	1445	1495	0.09% ± 0.03%	—
33	48.55	(+)‐delta‐Cadinene	C_15_H_24_	483‐76‐1	1472	1524	0.09% ± 0.03%	—
34	48.74	β‐Sesquiphellandrene	C_15_H_24_	20307‐83‐9	1484	1524	0.02% ± 0.01%	—
Alcohols								
36	26.52	Eucalyptol	C_10_H_18_O	470‐82‐6	1019	1032	0.17% ± 0.01%	0.27% ± 0.01%
37	38.67	Sabinene hydrate	C_10_H_18_O	546‐79‐2	1456	1451	—	0.19% ± 0.00%
38	41.53	Linalool	C_10_H_18_O	78‐70‐6	1495	1507	—	1.23% ± 0.08%
39	42.05	cis‐Sabinene hydroxide	C_10_H_18_O	17699‐16‐0	1503		—	0.14% ± 0.00%
40	42.60	cis‐p‐Menth‐2‐en‐1‐ol	C_10_H_18_O	29803‐81‐4	1253	1140	0.06% ± 0.00%	0.42% ± 0.01%
41	43.24	Fenchol	C_10_H_18_O	1632‐73‐1	1519		—	0.21% ± 0.01%
42	43.97	4‐Terpineol, (−)—	C_10_H_18_O	20126‐76‐5	1273	1182	1.71% ± 0.03%	9.53% ± 0.52%
43	45.67	(−)‐trans‐Pinocarveol	C_10_H_16_O	547‐61‐5	1298	1139	0.28% ± 0.01%	0.32% ± 0.02%
44	45.96	Isoborneol	C_10_H_18_O	124‐76‐5	1309	1157	1.27% ± 0.03%	—
45	46.58	L‐α‐Terpineol	C_10_H_18_O	10482‐56‐1	1348	1190	0.50% ± 0.03%	1.65% ± 0.11%
46	46.92	endo‐Borneol	C_10_H_18_O	507‐70‐0	1369	1170	16.18% ± 0.39%	0.40% ± 0.01%
47	46.97	Isopinocampheol	C_10_H_18_O	27779‐29‐9	1572		—	0.22% ± 0.01%
48	47.31	p‐Mentha‐1,5‐dien‐8‐ol	C_10_H_16_O	1686‐20‐0	1394	1167	0.19% ± 0.01%	0.14% ± 0.01%
49	47.94	trans‐Piperitol	C_10_H_18_O	16721‐39‐4	1585	1675	—	0.74% ± 0.05%
50	48.84	p‐Menth‐2‐en‐7‐ol, cis—	C_10_H_18_O	19898‐86‐3	1598		—	0.07% ± 0.02%
51	49.51	(−)‐Myrtenol	C_10_H_16_O	19894‐97‐4	1533	1213	0.69% ± 0.06%	1.63% ± 0.04%
52	50.44	(−)‐cis‐Carveol	C_10_H_16_O	1197‐06‐4	1592	1229	0.06% ± 0.09%	—
53	50.60	2‐Caren‐4‐ol	C_10_H_16_O	6617‐35‐2	1786		—	0.03% ± 0.00%
54	50.73	p‐Cymen‐8‐ol	C_10_H_14_O	1197‐01‐9	1790	1802	—	0.35% ± 0.02%
55	51.38	trans‐Myrtanol	C_10_H_18_O	53369‐17‐8	1819		—	0.04% ± 0.00%
Ketones								
35	20.08	(+)‐Camphor	C10H16O	464‐49‐3	887	1144	0.44% ± 0.04%	—
56	36.03	Fenchone	C_10_H_16_O	1195‐79‐5	1158	1096	0.19% ± 0.00%	—
57	38.13	β‐Thujone	C_10_H_16_O	471‐15‐8	1188	1114	0.02% ± 0.00%	0.03% ± 0.00%
58	41.38	1,4‐Dimethyl‐3‐cyclohexenyl methyl ketone	C_10_H_16_O	43219‐68‐7	1493	1505	—	0.12% ± 0.01%
59	42.61	3‐Pinanone	C_10_H_16_O	15358‐88‐0	1510	1511	—	1.39% ± 0.04%
60	43.04	Sabinone	C_10_H_14_O	67690‐48‐6	1516		—	0.04% ± 0.00%
61	43.32	α‐Pinocarvone	C_10_H_14_O	30460‐92‐5	1520	1539	—	0.40% ± 0.01%
62	45.31	Sabine Ketone	C_9_H_14_O	513‐20‐2	1548	1606	0.06% ± 0.00%	0.10% ± 0.00%
Aldehydes								
63	37.03	α‐Campholenal	C_10_H_16_O	4501‐58‐0	1172	1125	0.02% ± 0.00%	0.30% ± 0.01%
64	44.97	α‐Thujenal	C_10_H_14_O	57129‐54‐1	1543		—	0.07% ± 0.01%
65	45.19	Myrtenal	C_10_H_14_O	564‐94‐3	1291	1193	0.18% ± 0.01%	0.33% ± 0.01%
66	47.61	Citral	C_10_H_16_O	5392‐40‐5	1580	1714	—	0.41% ± 0.05%
67	49.99	Terpinen‐7‐al	C_10_H_14_O	1197‐15‐5	1563	1283	0.02% ± 0.01%	0.07% ± 0.01%
Esters								
68	43.76	(−)‐Isopinocampheol, acetate	C_12_H_20_O_2_	1000462‐98‐1	1527		—	0.28% ± 0.04%
69	43.78	Bornyl acetate	C_12_H_20_O_2_	76‐49‐3	1270	1287	3.57% ± 0.32%	—
70	46.41	trans‐Geranic acid methyl ester	C_11_H_18_O_2_	1189‐09‐9	1337	1322	0.03% ± 0.01%	0.02% ± 0.00%
71	46.51	Myrtenyl acetate	C_12_H_18_O_2_	1079‐01‐2	1565		—	0.88% ± 0.12%
72	46.69	Methyl myrtenate	C_11_H_16_O_2_	30649‐97‐9	1568	1670	—	0.85% ± 0.09%
73	49.09	Sabinyl Acetate	C_12_H_18_O_2_	3536‐54‐7	1738	1615	—	0.03% ± 0.00%
Others								
74	29.32	p‐Cymene	C_10_H_14_	99‐87‐6	1060	1022	2.03% ± 0.02%	17.29% ± 0.37%
75	35.35	m‐tert‐Butylphenol	C_10_H_14_O	585‐34‐2	1411	1415	—	0.03% ± 0.00%
76	36.38	Perillene	C_10_H_14_O	539‐52‐6	1425	1431	—	0.03% ± 0.00%
77	40.40	Dihydroedulan II	C_13_H_22_O	41678‐32‐4	1480	1492	—	0.16% ± 0.02%
78	40.76	theaspirane	C_13_H_22_O	36431‐72‐8	1485	1487	—	0.05% ± 0.01%

*Note:* KI values are experimental (polar column); KI* values are from literature (non‐polar columns). Differences between KI and KI* for the same compound arise from column polarity, with the magnitude related to compound polarity. Such differences are normal in volatile oil analysis.

Abbreviation: RT: retention time.

### 3.3 Differential Analysis of Chemical Components in Volatile Oils From Fruit and Stem

Principal component analysis (PCA) was performed on all identified components and their relative abundances to discern compositional differences between the two volatile oils. PCA is a classic multivariate statistical method that reduces the dimensionality of high‐dimensional data via orthogonal transformation, extracting principal components that capture the maximum variance in the dataset (Li et al. 2019). This approach enables the intuitive visualization of complex compositional data, where sample proximity in the reduced‐dimensional space reflects chemical profile similarity.

**FIGURE 3:**
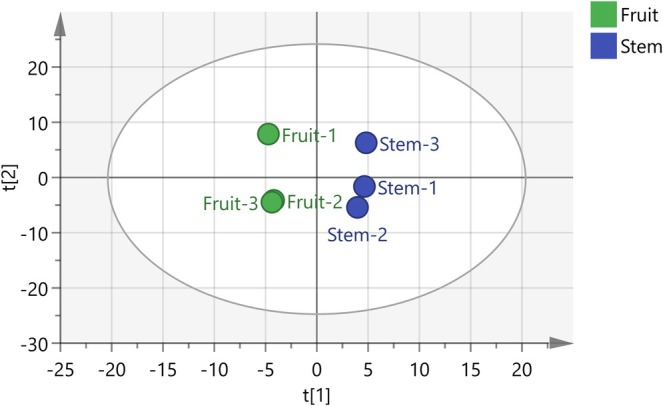
PCA score plot of volatile oil components from *W. villosa* fruit and stem.

The PCA results are shown in Figure 3. In the two‐dimensional score plot defined by the first two principal components (PC1 and PC2), fruit and stem volatile oil samples were clearly separated into distinct clusters. PC1 and PC2 cumulatively explained 85.5% of the variance, with individual contributions of 50.3% and 35.2%, respectively. The distribution of samples in separate regions along both principal component axes indicates significant chemical compositional differences between the two volatile oils.

### 3.4 Identification of Differential Markers Using OPLS‐DA

Orthogonal partial least squares‐discriminant analysis (OPLS‐DA) was applied to the compositional data to further elucidate volatile component differences between the fruit and stem volatile oils. This supervised multivariate method reduces data dimensionality for visualization and is particularly suitable for discriminant analysis and class prediction (Guo et al. 2025).

**FIGURE 4:**
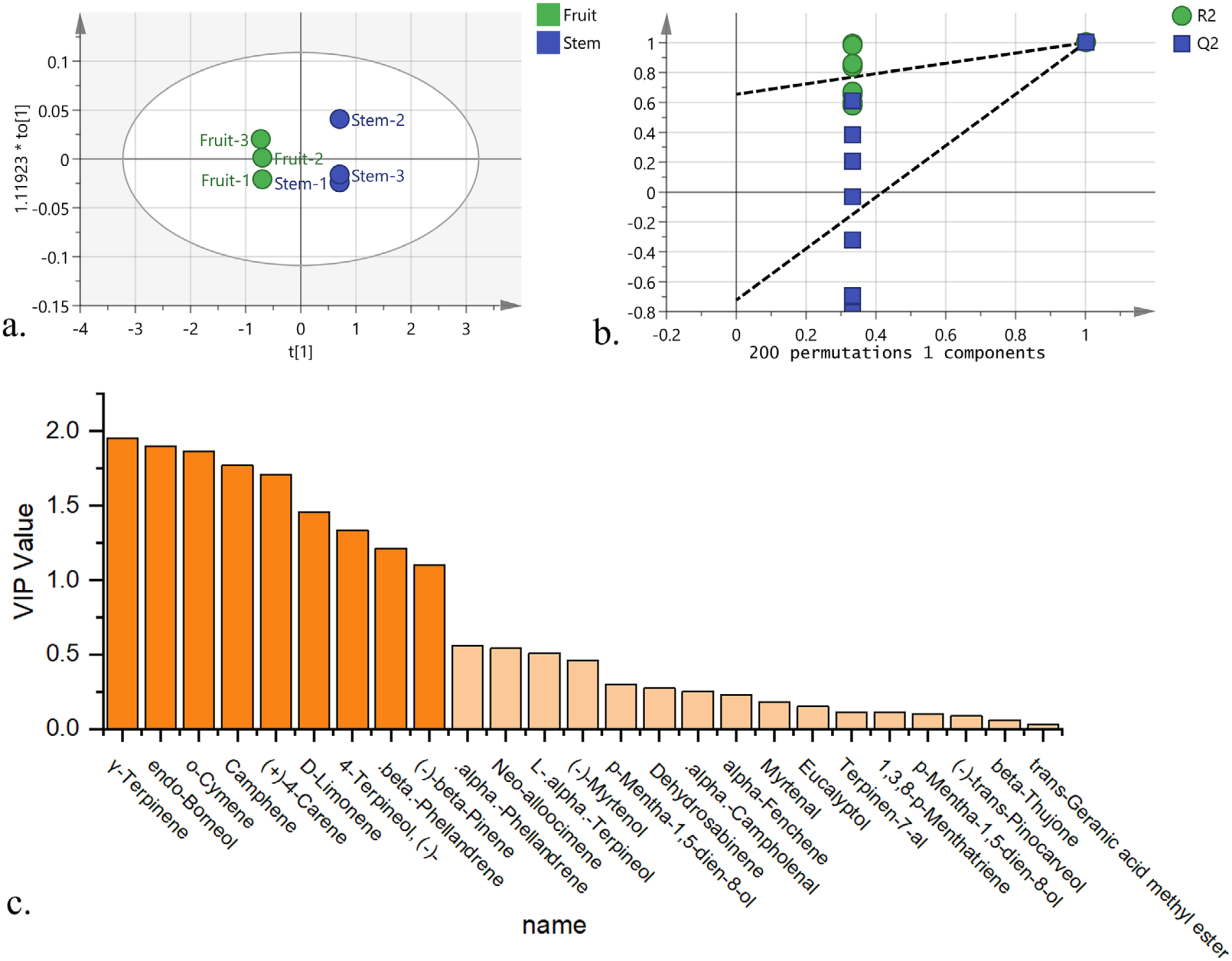
Orthogonal Partial Least Squares Discriminant Analysis (OPLS‐DA) of volatile oils from *W. villosa* fruit and stem: (a) score plot; (b) permutation test results; (c) VIP scores.

The OPLS‐DA score plot (Figure 4a) showed clear and complete separation between the fruit and stem sample groups, with no overlap, confirming significant differences in their chemical profiles. The model exhibited strong performance, with parameters *R* ^2^ *X* = 0.998, *R* ^2^ *Y* = 1.00, and *Q* ^2^ = 1.00. An *R* ^2^ *X* value close to 1 indicates a high proportion of variance explained by the model, while *Q* ^2^ > 0.5 generally signifies good predictive ability (Zhang et al. 2019). These parameters indicate that the model effectively discriminates the volatile oil compositions of the two sample groups.

A permutation test (*n* = 200 permutations, Figure 4b) was performed to verify model reliability, yielding an *R* ^2^ intercept of 0.655 and a *Q* ^2^ intercept of −0.723. A *Q* ^2^ intercept below −0.05 generally indicates no overfitting in some reference, however our result demonstrates good model reliability and stability. Notably, the small sample size (*n* = 3 per group) may overestimate *Q* ^2^, but the negative intercept from the permutation test still supports model validity. Differential components were screened based on variable importance in projection (VIP) scores, which reflect each variable's contribution to the classification model. Variables with VIP > 1 are typically considered significant discriminators (Ran et al. 2025). Applying a threshold of VIP > 1 and statistical significance (*p* < 0.05), nine key differential components were identified. Ranked by descending VIP score, these were γ‐terpinene, borneol, p‐cymene, camphene, (+)‐4‐carene, D‐limonene, (−)‐4‐terpineol, β‐phellandrene, and (−)‐β‐pinene (Figure 4c).

### 3.5 Evaluation of Antioxidant Activity of Volatile Oils From Fruit and Stem

#### 3.5.1 DPPH Radical Scavenging Activity

**FIGURE 5:**
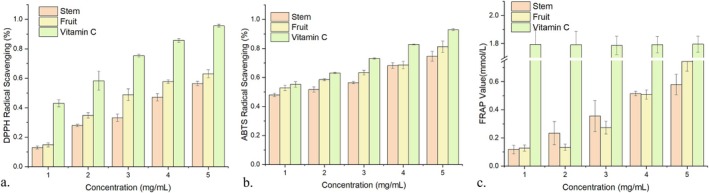
Differences in antioxidant activities of volatile oils from *W. villosa* fruit and stem (a) DPPH radical scavenging rate; (b) ABTS radical scavenging rate; (c) total antioxidant capacity.

In the DPPH radical scavenging assay (Figure 5a), the scavenging rates of both volatile oils increased progressively with rising concentrations in the range of 1–5 mg/mL. At 5 mg/mL, the fruit volatile oil achieved a scavenging rate of 63.00%, compared to 14.80% at 1 mg/mL, representing a 48.20% increase. The stem volatile oil reached 56.40% at 5 mg/mL, up from 13.00% at 1 mg/mL. The IC_50_ value of the fruit volatile oil (2.13 mg/mL, 95% CI: 2.06–2.20 mg/mL) was slightly lower than that of *V*_*c*_ (2.46 mg/mL, 95% CI: 2.30–2.63 mg/mL), indicating stronger radical scavenging activity, whereas the stem volatile oil showed weaker activity (IC_50_ = 3.83 mg/mL, 95% CI: 3.69–3.96 mg/mL). By comparison, the IC_50_ of Amomum tsao‐ko volatile oil was reported as 5.27 mg/mL (Cui et al. 2017), substantially higher than those of both volatile oils, suggesting inferior activity. Nevertheless, at 5 mg/mL, *V*_*c*_ exhibited a much higher scavenging rate (95.71%). Collectively, both the fruit and stem volatile oils possess moderate DPPH radical scavenging activity, with the fruit volatile oil being superior to the stem volatile oil.

#### 3.5.2 ABTS Radical Scavenging Activity

In the ABTS radical scavenging assay (Figure 5b), the scavenging rates of both volatile oils increased with rising concentrations (1–5 mg/mL). The scavenging activity of the fruit volatile oil increased from 52.74% to 81.28%. The stem volatile oil showed a similar trend, increasing from 48.00% to 74.67%. The IC_50_ values were 2.65 mg/mL (95% CI: 2.55–2.77 mg/mL) for the fruit volatile oil and 2.83 mg/mL (95% CI: 2.74–2.93 mg/mL) for the stem volatile oil. In comparison with literature reports, the IC_50_ of nutmeg ( *Myristica fragrans* ) volatile oil was 27.90 mg/mL (Sander et al. 2024), whereas that of galangal ( *Alpinia officinarum* ) volatile oil was 1.31 mg/mL (Xie et al. 2023), indicating that the ABTS scavenging capacity of the fruit and stem volatile oils fell between these two values. Compared with the positive control *V*_*c*_, which exhibited a scavenging rate of 95.72% at 5 mg/mL and an IC_50_ of 2.40 mg/mL (95% CI: 2.36–2.44 mg/mL), both volatile oils showed lower scavenging efficiency. In conclusion, although the fruit and stem volatile oils possess clear ABTS radical scavenging activity, their efficacy remains inferior to that of *V*_*c*_.

#### 3.5.3 Total Antioxidant Capacity (FRAP Assay)

The total antioxidant capacity of the fruit and stem volatile oils was evaluated using the FRAP assay (Figure 5c). The FRAP values of both volatile oils increased in a concentration‐dependent manner from 1 to 5 mg/mL. Specifically, the FRAP value of the fruit volatile oil increased from 0.13 to 0.74 mmol/L, representing a 4.69‐fold increase, while that of the stem volatile oil increased from 0.12 to 0.58 mmol/L, a 3.83‐fold increase. The IC_50_ values for the fruit and stem volatile oils were 3.78 mg/mL (95% CI: 3.45–4.14 mg/mL) and 3.80 mg/mL (95% CI: 3.55–4.07 mg/mL), respectively, indicating comparable ferric reducing capacity at the 50% level. In comparison, the IC_50_ of *Cladanthus mixtus* essential oil was reported to be 2.01 mg/mL (El Kamari et al. 2025), suggesting that both volatile oils possess weaker FRAP activity. Compared with the positive control *V*_*c*_, which exhibited a FRAP value of 1.80 mmol/L at 5 mg/mL and an IC_50_ of 0.66 mg/mL (95% CI: 0.52–0.82 mg/mL), both volatile oils showed substantially lower antioxidant potency on a per‐concentration basis.

### 3.6 Evaluation of In Vitro Antibacterial Activity of Volatile Oils From Fruit and Stem

**FIGURE 6:**
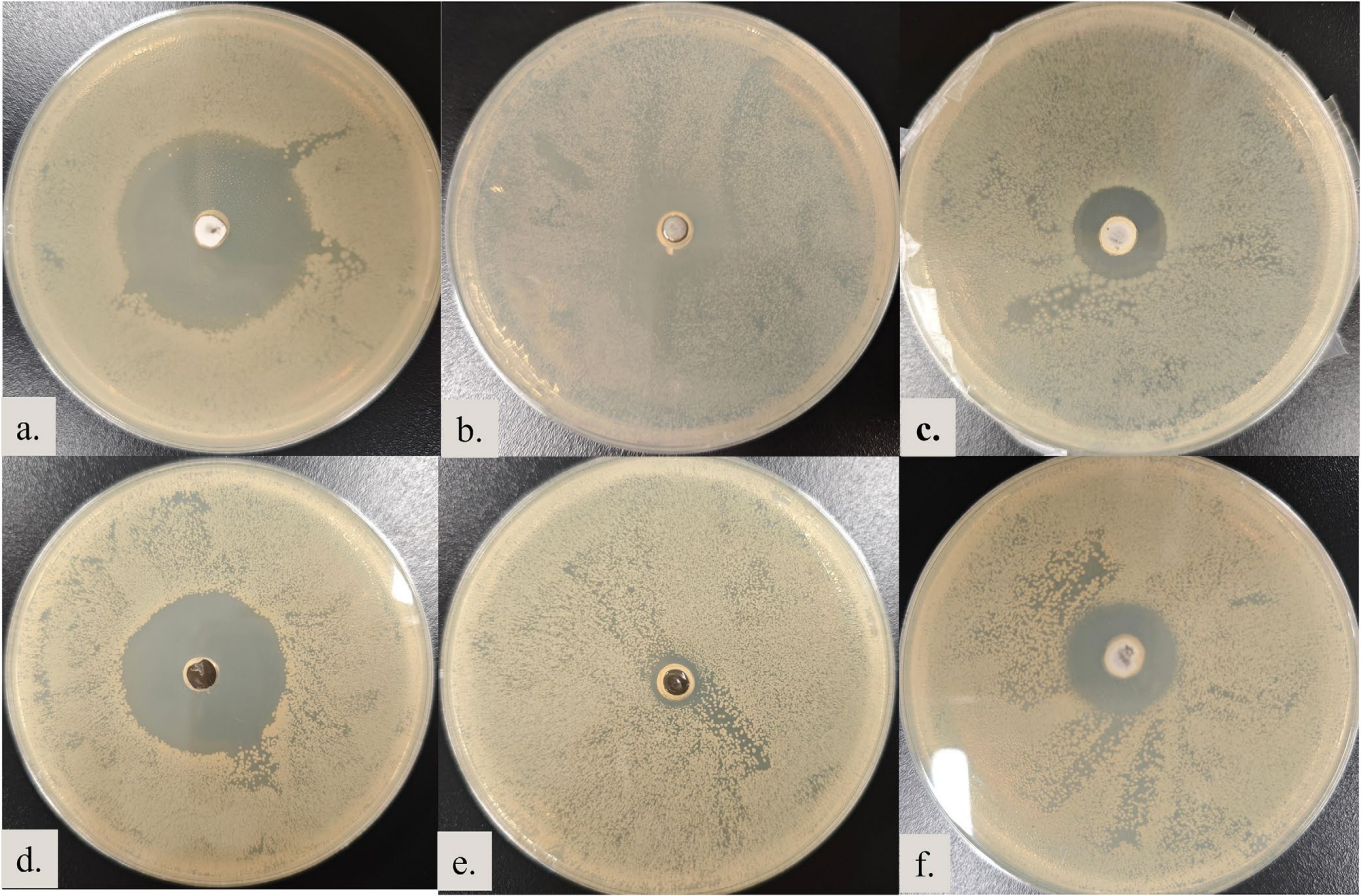
Antibacterial effects of volatile oils from *W. villosa* fruit and stem against *E. coli* and *S. aureus* (a) Ampicillin against *E. coli* ; (b) *W. villosa* fruit volatile oil against *E. coli* ; (c) *W. villosa* stem volatile oil against *S. aureus* ; (d) Penicillin against *S. aureus* ; (e) *W. villosa* fruit volatile oil against *S. aureus* ; (f) *W. villosa* stem volatile oil against *S. aureus* .

Antibacterial activity was evaluated by measuring inhibition zone diameter, a direct and quantitative metric of efficacy. Antibacterial sensitivity was classified according to established criteria (Chen et al. 2026) as follows: insensitive (no inhibition zone), mildly sensitive (< 10 mm), moderately sensitive (10–15 mm), highly sensitive (15–20 mm), and extremely sensitive (≥ 20 mm). As shown in Figure 6, the positive controls exhibited significant antibacterial activity: ampicillin produced an inhibition zone of 31.84 ± 0.91 mm against *E. coli* (Figure 6a), and penicillin produced a zone of 36.32 ± 1.03 mm against *S. aureus* (Figure 6d). In contrast, the fruit volatile oil showed weak antibacterial effects, with an inhibition zone of 7.04 ± 0.55 mm against *S. aureus* (Figure 6e) and no obvious zone against *E. coli* (Figure 6b). Conversely, the stem volatile oil exhibited clear antibacterial activity against both pathogens, with inhibition zones of 20.98 ± 2.03 mm against *S. aureus* (Figure 6f) and 15.44 ± 1.13 mm against *E. coli* (Figure 6c). In summary, the positive controls ampicillin and penicillin exhibited high sensitivity against *E. coli* and *S. aureus* , respectively. The stem volatile oil was exhibited extreme sensitivity against *S. aureus* and highly sensitive to *E. coli* . By comparison, the inhibition zones of *Amomum subulatum* volatile oil were 1.1 ± 0.2 mm against *S. aureus* and 1.0 ± 0.2 mm against *E. coli* (Prashant and Nagaiyan 2021), which are substantially smaller than those of both *W. villosa* oils reported in this study. Collectively, these results indicate that stem volatile oil possesses strong antibacterial activity against *E. coli* and *S. aureus* , while the fruit volatile oil shows mild activity against *S. aureus* and no detectable activity against *E. coli* .

### 3.7 Evaluation of In Vitro Hypoglycemic Activity of Fruit and Stem

#### 3.7.1 α‐Glucosidase Inhibitory Activity

**FIGURE 7:**
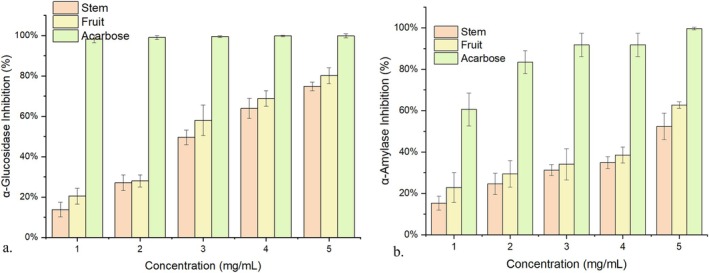
Inhibitory effects of volatile oils from *W. villosa* fruit and stem on α‐glucosidase and α‐amylase activities (a) α‐Glucosidase inhibition rate; (b) α‐Amylase inhibition rate.

α‐Glucosidase is a key carbohydrate‐digesting enzyme located in the brush border of the small intestine, and its activity directly affects the breakdown of dietary polysaccharides into absorbable monosaccharides (Guo et al. 2024). Therefore, inhibition of α‐glucosidase activity slows glucose release and absorption, representing an effective strategy for postprandial blood glucose control. In the in vitro hypoglycemic assay, the inhibitory effects of the fruit and stem volatile oils against α‐glucosidase were determined at concentrations of 1–5 mg/mL (Figure 7a). At 5 mg/mL, the inhibition rates of the fruit and stem volatile oils were 80.12% and 74.77%, respectively, while that of the positive control acarbose was 99.86%. The IC_50_ values were 3.16 mg/mL (95% CI: 2.97–3.37 mg/mL) for the fruit volatile oil, 3.38 mg/mL (95% CI: 3.28–3.47 mg/mL) for the stem volatile oil, and 1.74 mg/mL (95% CI: 1.72–1.78 mg/mL) for acarbose. Notably, the IC_50_ of *W. villosa* volatile oil from Baise, Guangxi, was reported to be 0.99 mg/mL (Wu et al. 2025), which is substantially highter than the two volatile oils in this study, possibly due to differences in geographical origin, extraction method, or sample processing. Overall, although both volatile oils in this study exhibited certain α‐glucosidase inhibitory activity, their potency was lower than that of acarbose.

#### 3.7.2 α‐Amylase Inhibitory Activity

**FIGURE 8:**
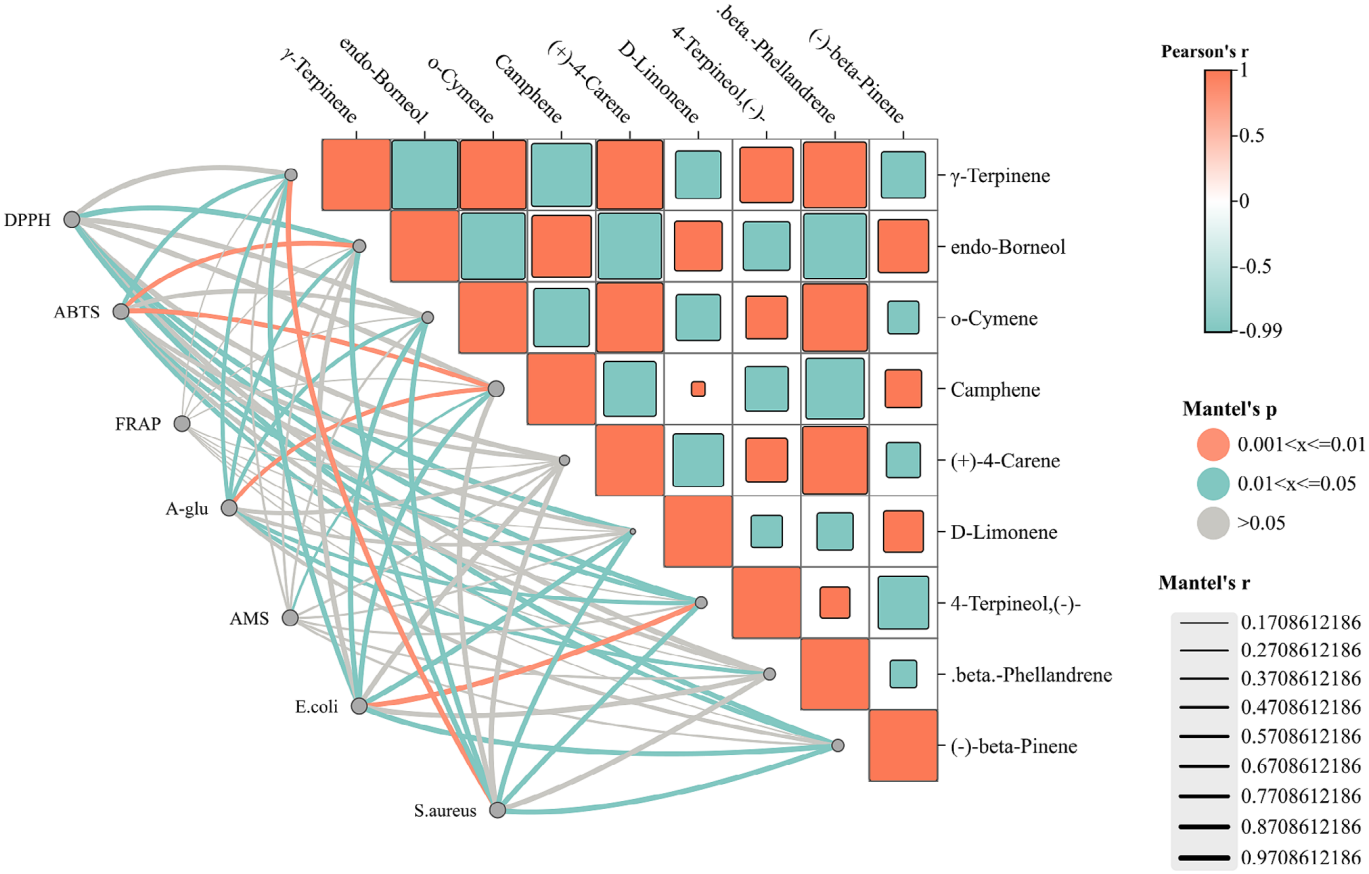
Correlation analysis between volatile components and biological activities.

α‐Amylase is a key digestive enzyme in the human body that hydrolyzes the α‐1,4 glycosidic bonds of ingested carbohydrates to produce glucose. Inhibiting α‐amylase activity reduces glucose absorption, thereby achieving a hypoglycemic effect (Yang et al. 2021). As shown in Figure 8b, the inhibitory effects of both volatile oils against α‐amylase increased with rising concentrations (1–5 mg/mL), showing a positive correlation. At 5 mg/mL, the inhibition rate of the fruit volatile oil was 62.69%, while that of the stem volatile oil was slightly lower (52.40%), compared with 99.66% for the positive control acarbose. The IC_50_ values were 3.73 mg/mL (95% CI: 3.35–4.15 mg/mL) for the fruit volatile oil, 4.00 mg/mL (95% CI: 3.78–4.23 mg/mL) for the stem volatile oil, and 2.14 mg/mL (95% CI: 2.05–2.24 mg/mL) for acarbose. In comparison, the IC_50_ values of *Wurfbainia schmidtii* leaf and rhizome volatile oils were reported to be 1.85 and 3.83 mg/mL, respectively (Trung et al. 2024), with the rhizome oil showing activity comparable to that of the fruit volatile oil in this study. Overall, although the IC_50_ values of the two volatile oils in this study differed substantially from that of acarbose, they still exhibited moderate inhibitory activity.

### 3.8 Correlation Analysis Between Chemical Components and Biological Activities of Fruit and Stem Volatile Oils

To elucidate the chemical basis for the differences in bioactivity between fruit and stem volatile oils, Mantel tests were performed to analyze the correlations between the major common components and various bioactivity indicators (Gao et al. 2025) (Figure 8). The figure presents raw, uncorrected correlation coefficients for exploratory reference only. Based on the raw correlation coefficients: DPPH scavenging positively correlated with γ‐terpinene, borneol, p‐cymene, camphene, (+)‐4‐carene, D‐limonene, β‐phellandrene, and (−)‐β‐pinene, but negatively correlated with (−)‐4‐terpineol. ABTS radical scavenging capacity was positively correlated with γ‐terpinene, borneol, camphene, (−)‐4‐terpineol, and (−)‐β‐pinene. FRAP reducing power showed no correlation with any of the components. α‐Glucosidase inhibitory activity was positively correlated with γ‐terpinene, borneol, p‐cymene, camphene, and β‐phellandrene. α‐Amylase inhibitory activity was positively correlated only with (−)‐4‐terpineol. *E. coli* inhibition positively correlated with γ‐terpinene, p‐cymene, (−)‐4‐terpineol, and (−)‐β‐pinene, while inhibition against *S. aureus* inhibition positively correlated with γ‐terpinene, borneol, p‐cymene, D‐limonene, (−)‐4‐terpineol, and (−)‐β‐pinene. However, after Benjamini–Yekutieli (BY) correction, no correlations remained significant (q > 0.05), suggesting weak associations between chemical components and bioactivities, possibly due to limited sample size.

## 4 Discussion

This study systematically compared the chemical composition and biological activities of volatile oils from the fruit and stem of *W. villosa* , revealing significant part‐specific differences. The yield of fruit volatile oil (1.87%) was substantially higher than that of the stem volatile oil (0.13%). Forty‐six and fifty‐nine components were identified in the fruit and stem volatile oils, respectively, with 27 common to both. The fruit volatile oil was characterized by high‐content components including α‐pinene, camphene, (−)‐β‐pinene, D‐limonene, and borneol, whereas the stem volatile oil was dominated by γ‐terpinene, p‐cymene, (+)‐4‐carene, and (−)‐4‐terpineol. OPLS‐DA further identified nine key differential markers, confirming significant chemical differences between the two volatile oils. The volatile oil yield from *W. villosa* by hydrodistillation was 1.50%, with bornyl acetate and borneol contents ranging from 30.01% to 38.18% and 9.21% to 14.68%, respectively (Bui et al. 2022). The yield and major component contents determined in this study differed from the above results, which may be attributed to factors such as germplasm quality, growth environment, storage conditions, and extraction process.

Differences in chemical composition may underlie the variations in bioactivity between the two volatile oils. Although the Mantel test was not statistically significant after multiple correction (*q* > 0.05), the trend from the correlation analysis indicated that characteristic components positively correlated with antioxidant and hypoglycemic activities with antioxidant and hypoglycemic activities—such as borneol (16.18%), D‐limonene (14.66%), (−)‐β‐pinene (14.93%), and camphene (14.53%)—were present at substantially higher concentrations in the fruit volatile oil than in the stem volatile oil. Previous studies have shown that borneol exhibits antioxidant and antidiabetic properties (Cahyana et al. 2023); D‐limonene has antioxidant (Venkatesan et al. 2025) and antihyperglycemic effects (Kumar et al. 2020); α/β‐pinene isomers exhibit antioxidant and hypoglycemic activities (Helia et al. 2023; Santos et al. 2022; Soares et al. 2023); and camphene has shown similar efficacy (Alwan et al. 2025; Khan et al. 2025). Taken together, these findings tentatively suggest that the superior antioxidant and hypoglycemic activities of the fruit volatile oil relative to the stem volatile oil are potentially attributable to the synergistic contribution of the aforementioned components.

Conversely, the stem volatile oil demonstrated superior antibacterial activity. Correlation analysis indicated that this activity was closely associated with components such as γ‐terpinene, p‐cymene, and (−)‐4‐terpineol. Our data showed that the contents of these key antibacterial components were generally higher in the stems: γ‐terpinene 17.78%, p‐cymene 17.29%, and (−)‐4‐terpineol 9.53%. Previous studies have shown that, γ‐terpinene has antibacterial properties (Sousa et al. 2022; Guo et al. 2020); p‐cymene, which is widely present in plant volatile oils, demonstrates good antibacterial activity (Sousa et al. 2022); and (−)‐4‐terpineol has been shown to enhance bacterial outer membrane permeability, prevent biofilm formation, and exert strong antibacterial effects (Cuong et al. 2011; Nair et al. 2025; Zhao et al. 2021). Therefore, the high abundance of γ‐terpinene, p‐cymene, and (−)‐4‐terpineol may collectively underlie the antibacterial activity of the stem volatile oil.

Together, the correlational trends and quantitative compositional data suggest that the differences in bioactivities between the volatile oils from distinct parts of *W. villosa* may be related to their specific chemical profiles. Although no statistically significant associations remained after multiple correction, these findings can be considered exploratory and warrant further validation. Additionally, this study has limitations, including the lack of in vivo validation and insufficient mechanistic exploration, which need to be addressed in future investigations.

Building on these findings, future research should focus on the formation mechanisms of key differential markers between fruits and stems, their bioactive targets, structure–activity relationships, and high‐value applications: First, based on the preliminary composition–activity trends, network pharmacology can be employed to construct a “component–target–pathway” multi‐dimensional network to predict the multi‐target integrative mechanisms, followed by validation through in vivo animal experiments. Second, focusing on the potential key differential components (e.g., borneol and D‐limonene in fruits; γ‐terpinene and (−)‐4‐terpineol in stems), dose–response studies and comparative activity evaluations of individual compounds and their combinations should be conducted to assess their efficacy contributions and interactions, thereby providing evidence for the characteristic active substance basis of each tissue (Hu et al. 2019; Shao et al. 2022). Third, given the significant differences in chemical profiles between different parts, chemometric methods should be established to enable accurate identification and quality control models, ensuring standardization and rationalization in resource utilization. Fourth, to elucidate the metabolic regulatory mechanisms underlying the differences in volatile oil composition between the fruits and stems, integrating multi‐omics technologies such as plant metabolomics, transcriptomics, and proteomics to explore the molecular basis of tissue‐specific chemical profiles, thereby providing theoretical clues for cultivation optimization or synthetic biology approaches to enhance target components. Collectively, these studies are expected to promote the whole‐plant and high‐value utilization of *W. villosa* resources, developing them into natural antioxidants, hypoglycemic adjuvants, and green antimicrobial agents.

## 5 Conclusion

This study revealed significant tissue‐dependent differences in chemical composition and bioactivity between the volatile oils of *W. villosa* fruits and stems. The fruit volatile oil, enriched in borneol, D‐limonene, pinene, and camphene, exhibited superior antioxidant and hypoglycemic activities, whereas the stem oil, enriched in γ‐terpinene, p‐cymene, and (−)‐4‐terpineol, demonstrated stronger antibacterial activity. The Mantel correlation trend, together with the quantitative compositional data, suggested that the observed differences in bioactivity were associated with the differential contents of their characteristic components. In summary, the fruit volatile oil shows potential for development as a natural antioxidant and hypoglycemic adjuvant, while the stem volatile oil holds promise as a green antimicrobial agent.

## Author Contributions

**Bangyu Lv:** writing – review and editing, supervision, resources. **Xingrui Nian:** methodology. **Yuancong Gu:** writing – review and editing, writing – original draft, supervision, data curation, resources. **Xinhe Yang:** writing – review and editing, funding acquisition, project administration, supervision. **Xinrui Xie:** methodology.

## Funding

This research was supported by 2023 Guangdong Province Science and Technology Innovation Special Fund (“Major Special Fund + Task List”) Project (SDZX2023038), 2024 Guangdong Provincial Science & Technology Support Program for the High‐Quality Development Project of 100 Counties, 1000 Towns, and 10,000 Villages (BQW2024009 and SBQW2024014) and program for scientific research start‐up funds of Guangdong Ocean University (360302062204).

## Conflicts of Interest

The authors declare no conflicts of interest.

## Data Availability

The data that support the findings of this study are available from the corresponding author upon reasonable request.
